# Supplementary material for: Natural genetic variation quantitatively regulates heart rate and dimension
Source: Nat Commun. 2025 Apr 30;16:4062. doi: 10.1038/s41467-025-59425-7 (PMC12044080; doi:10.1038/s41467-025-59425-7)
Supplement: Supplementary file 1 — Supplementary Information [file 41467_2025_59425_MOESM1_ESM.pdf]

Supplementary information

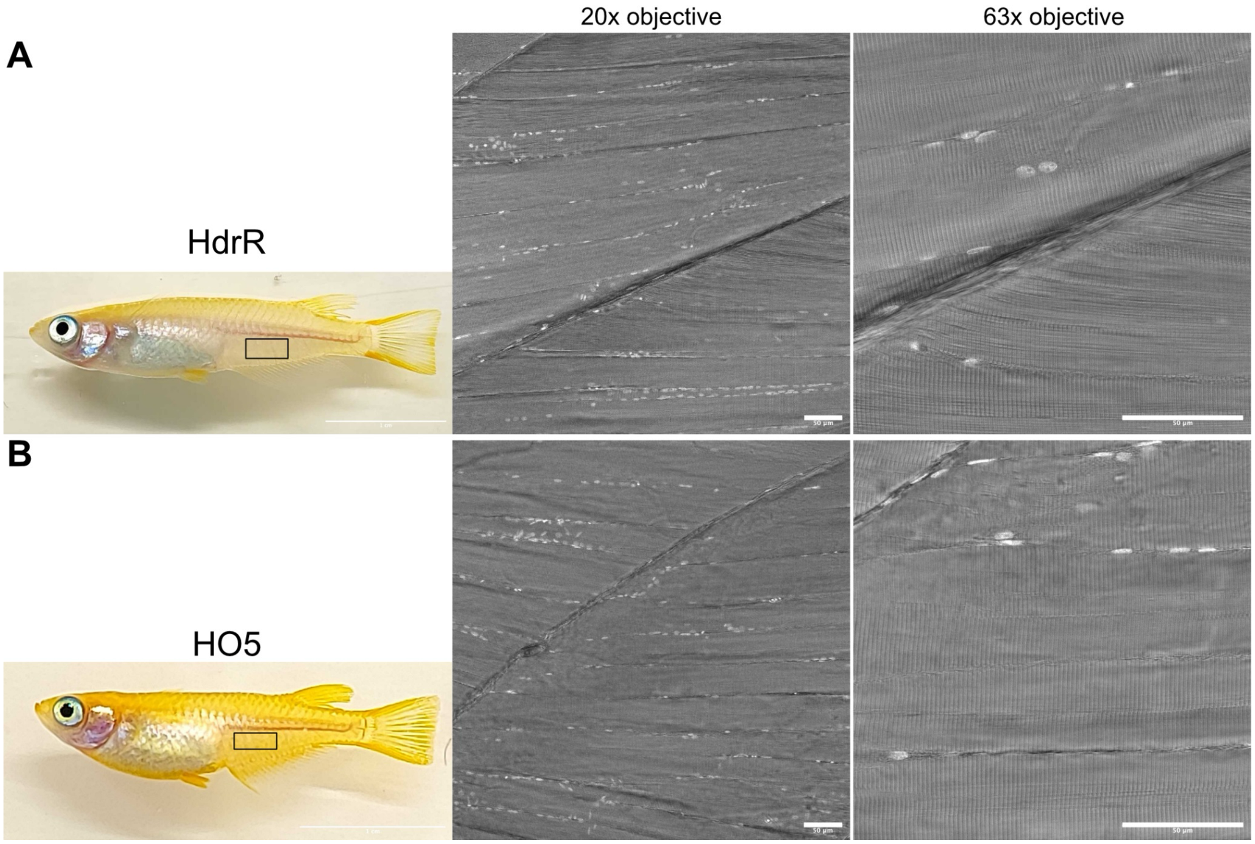

Fig. S1 Skeletal muscle structure in medaka HdrR and HO5 strains. Vibratome sections of adult HdrR (**A**) and HO5 **(B**) skeletal muscle. No structural differences were observed in bright-field images. DAPI-stained nuclei in white. Scale bar is 50 µm.

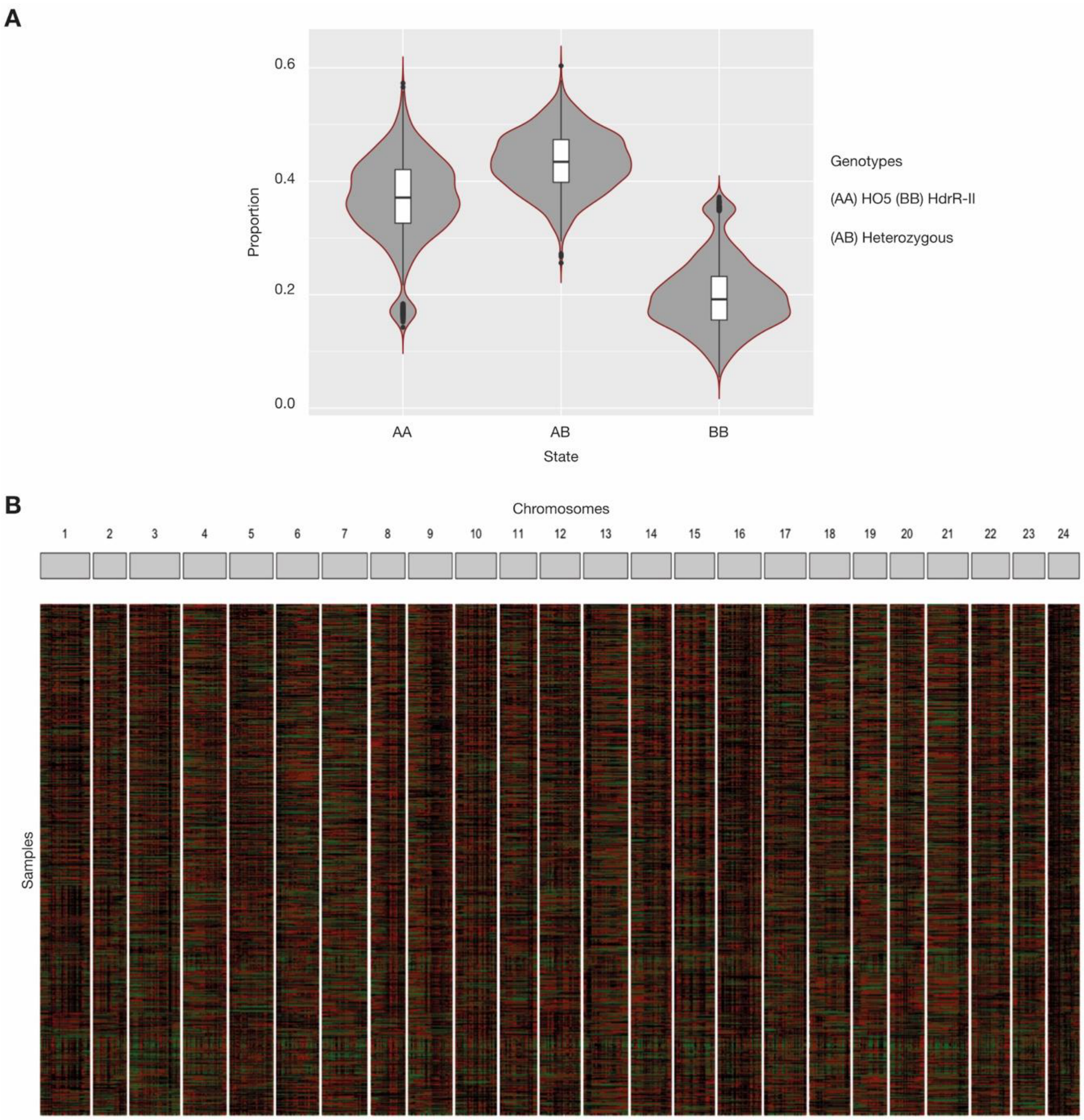

Fig. S2 Genotype proportions of the parental lines HdrR and HO5 in the sequenced F2 population. (**A**) Proportions of homozygous HO5 genotype (AA), homozygous HdrR genotype (BB) or heterozygous genotype (AB). (**B**) Genotypes and recombination block distribution for the F2 samples (rows) across the 24 chromosomes (columns). AA = black, BB = green, AB = red.

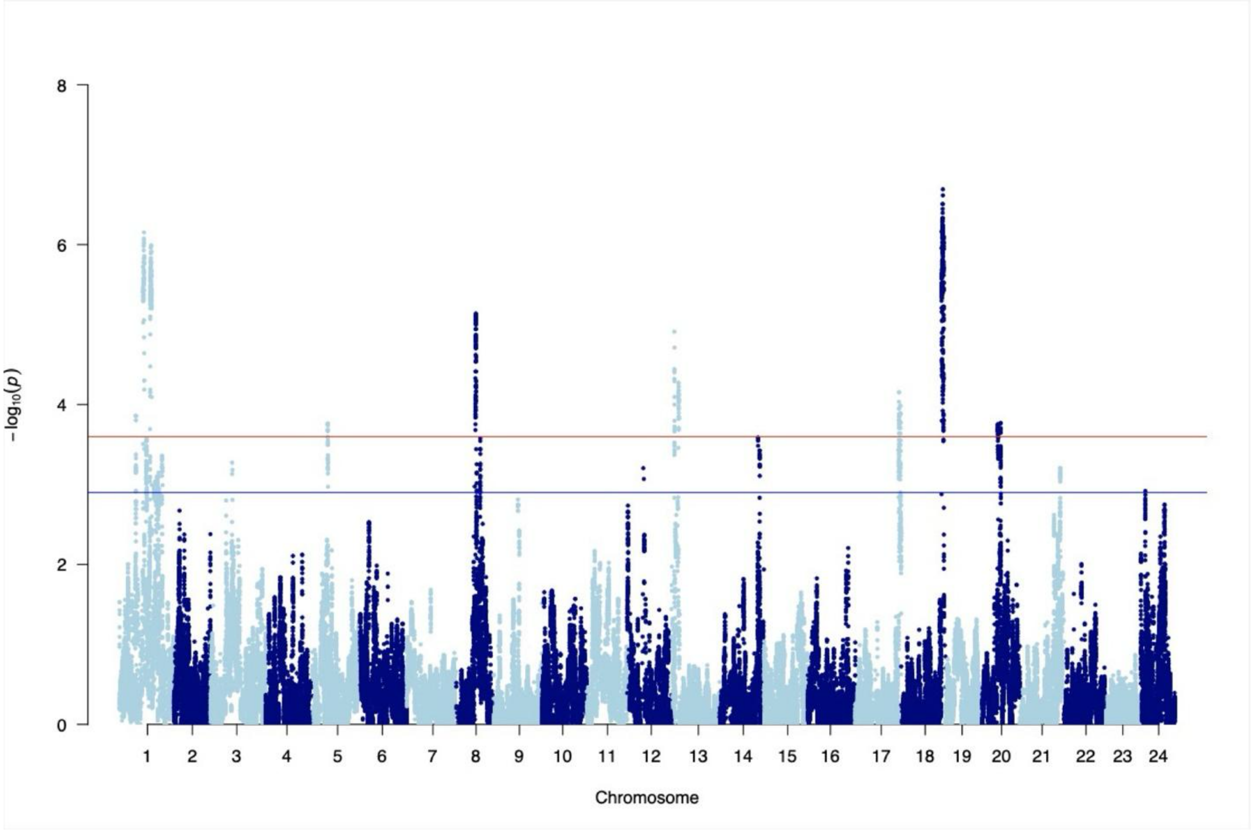

Fig. S3 Variance phenotype. Manhattan plot showing -log10 p values from the linear mixed model using the variance phenotype (mean absolute difference between repeated measurements on the same embryo across the 3 different temperatures).

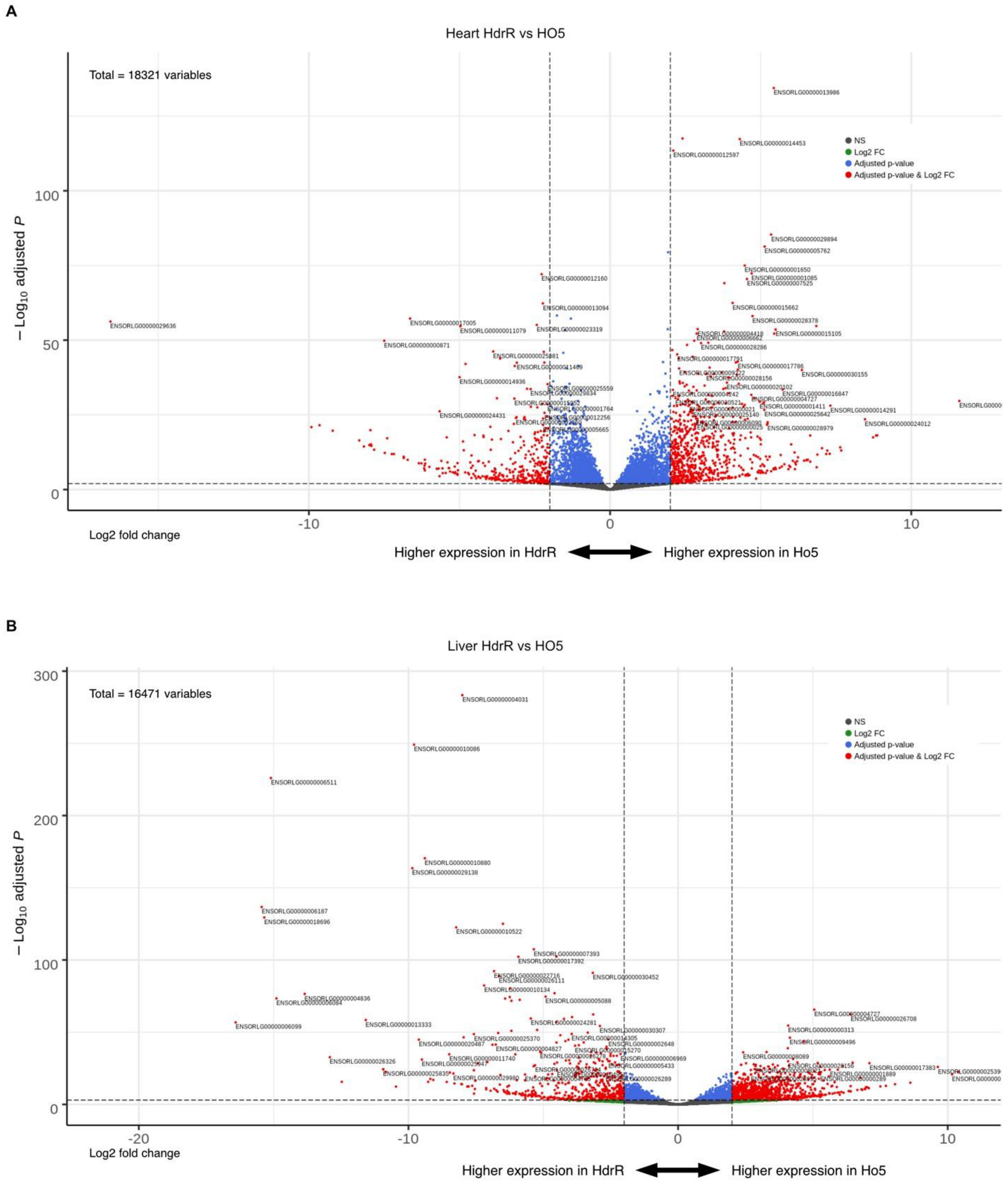

Fig. S4 Differentially expressed genes in HdrR versus HO5 heart and liver samples. (**A**, **B**) Volcano plots of HdrR and HO5 heart (A) and liver (B) comparative transcriptomics, -log10(adj-pvalue) compared with log2 Fold change.

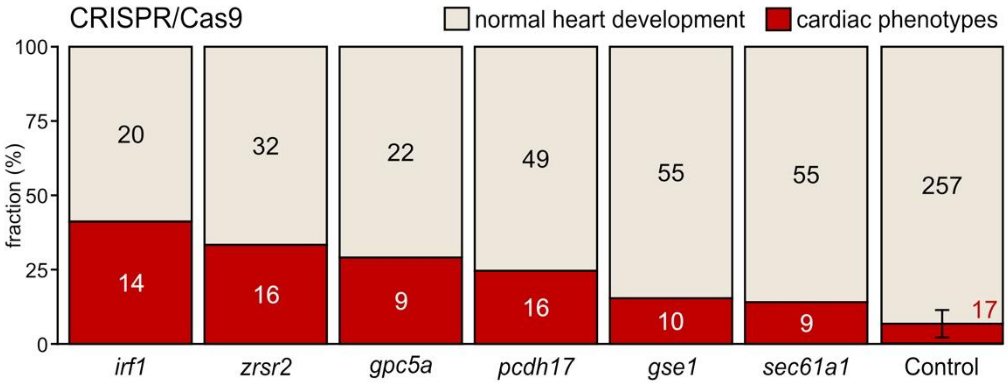

Fig. S5 Heart phenotype proportions in CRISPR-Cas9 knockout models of candidate genes. (**A**) Proportion (bars) and counts (values) of cardiac affected and normally developed embryos after CRISPR-Cas9-mediated knockout of indicated candidate genes versus control (mock injection) quantified at 4 days post fertilization (dpf). Phenotypic proportions of crispants were determined from 72 to 114 embryos and compared to 303 mock-injected control embryos. The mean and standard deviation of the control group was calculated based on 7 technical replicates.

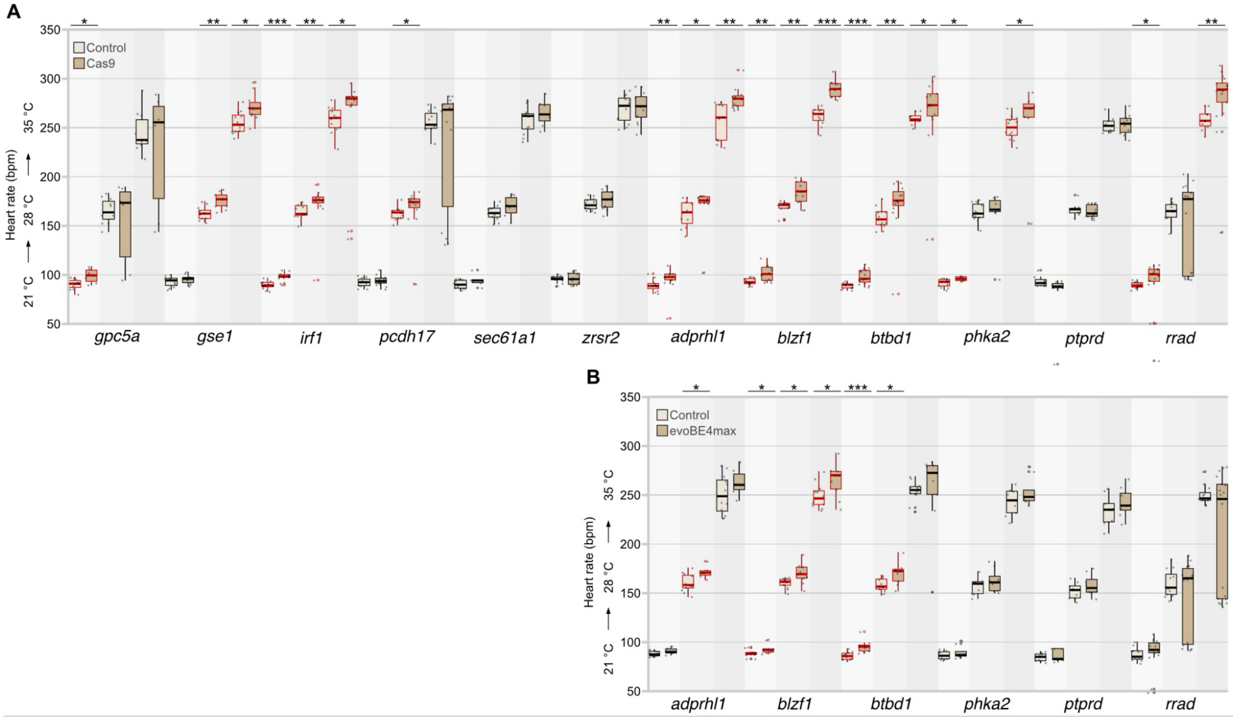

Fig. S6 Cardiac affected crispants and editants have altered heart rates 4 days post fertilization. Heart rate of crispants (n = 5 to 17 embryos) (**A**) and editants (n = 6 to 18 embryos) (**B**) with heart phenotypes in comparison to mock-injected control embryos with normally developed hearts (n = 8 to 12 embryos) at 21°C, 28°C, and 35°C (heart rate values are listed in data S2 and sample numbers in data S3). The significance of heart rate differences between the edited group and its corresponding control was assessed with the Wilcoxon test; *, p<0.05; **, p<0.01; ***, p<0.001 (p-values listed in data S4). Data is visualized as box plots (median+/- interquartile range between the 25th and 75th percentiles) and overlaid scatter plots of heart rate measurements; significant heart rate differences are highlighted in red. Data for heart rates in cardiac affected (this figure) and non-affected embryos (main Fig. 4) per gene were obtained per gene in a single experiment sharing the mock controls.

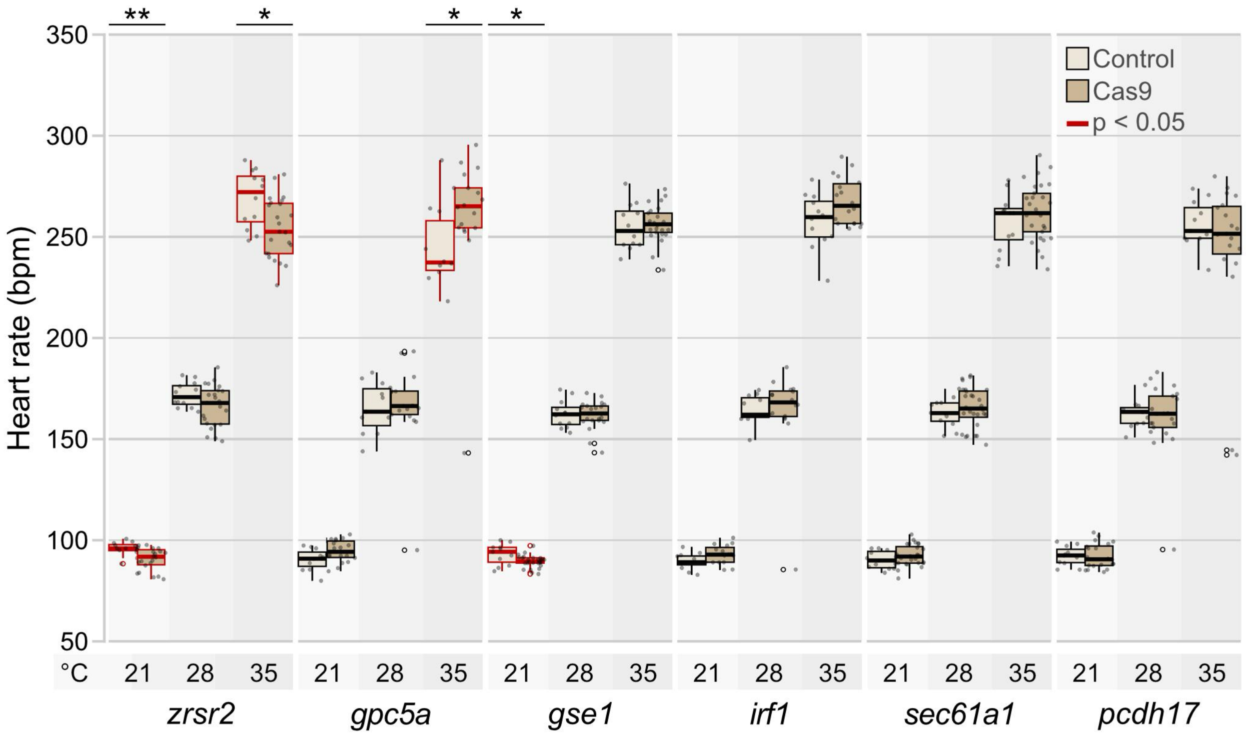

Fig. S7 Editing of candidate genes affect the heart rate level of medaka embryos. Heart rate distributions of morphologically normal CRISPR-Cas9-mediated gene edited embryos (n = 15 to 29 embryos) and mock-injected control embryos (n = 10 to 12 embryos) at 4 dpf at 21°C, 28°C, and 35°C (heart rate values are listed in data S2 and sample numbers in data S3).The significance of heart rate differences between the crispant group and its corresponding control was tested with the Wilcoxon test; *, p<0.05, **, p<0.01, ***, p<0.001 (p-values for all comparison groups are listed in data S4). Data is visualized as box plots (median+/- interquartile range between the 25th and 75th percentiles) and overlaid scatter plots of heart rate measurements; *zrsr2* crispants and *gse1* crispants show a temperature-dependent significant decrease in heart rate, in contrast, *gpc5a* crispants show a significant increase in heart rate at 35°C.

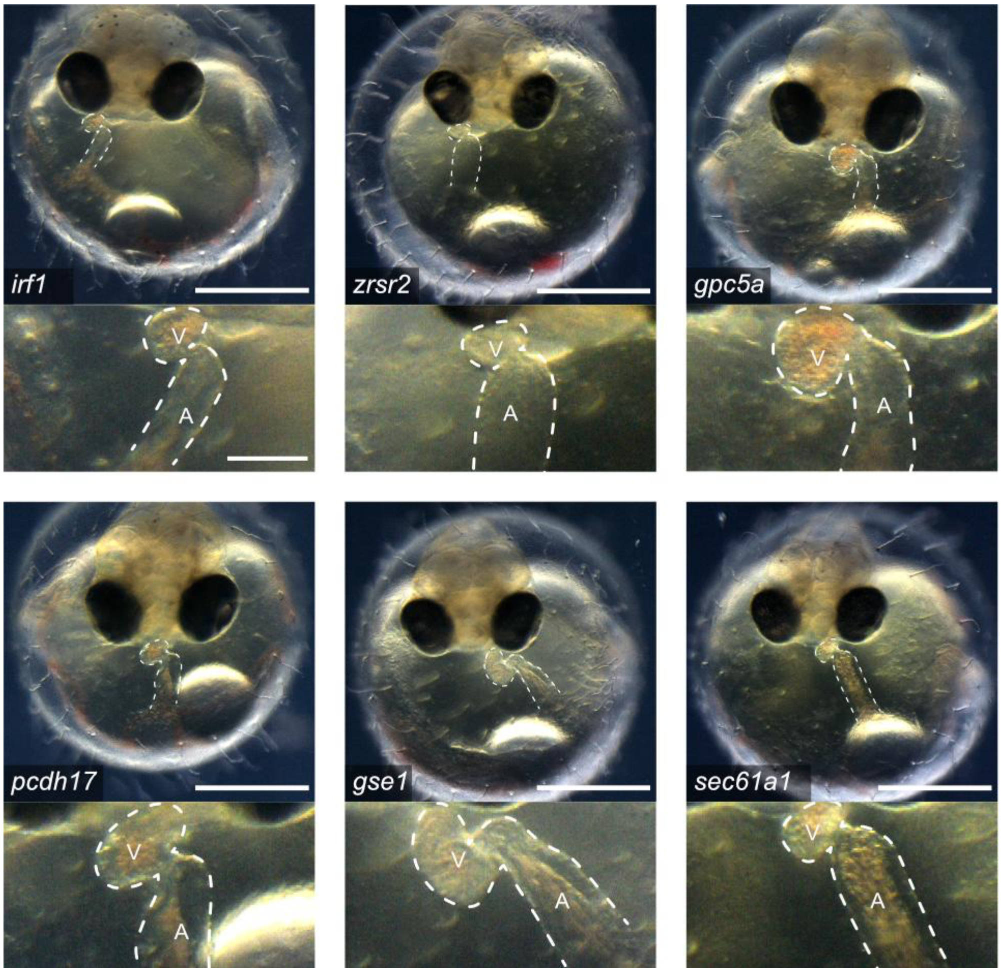

Fig. S8 Morphological heart phenotypes in CRISPR-Cas9 knockout models of candidate genes. Cardiac phenotypes of knockout embryos for the six candidate genes targeted with CRISPR-Cas9 at 4 dpf; bright-field overview of the injected specimen (top; scale bar, 500 µm), close-up image of the heart (bottom; scale bar, 125 µm). cf. movie S3.

Table S1 Numbers of embryos scored (n) in Fig. 1B for each strain and point in time. Fluctuations in the sample sizes are due to random embryonic movements during imaging that can obscure the heart and preclude measurement; embryos that have already hatched (swimming) have been excluded resulting in a drop of n at 148 hpf and 152 hpf.

Age (hpf)	n HdrR	n HNI F86	n HO5	n Cab	n Kaga
40	14	14	18	15	15
44	18	17	18	18	15
48	18	17	18	17	15
52	18	18	18	18	15
56	18	18	18	18	15
60	18	18	17	18	15
64	18	17	18	18	15
68	18	18	18	18	15
72	18	18	18	18	15
76	18	18	16	18	15
80	18	16	12	16	14
84	18	18	18	18	15
88	18	18	16	17	15
92	18	18	15	17	13
96	18	18	18	18	15
100	18	17	15	18	15
104	18	13	16	16	10
108	16	16	17	17	15
112	18	15	18	18	15
116	17	14	17	18	15
120	18	15	16	17	15
124	17	14	17	17	11
128	14	13	18	16	14
132	17	13	18	17	11
136	18	13	18	18	14
140	16	12	16	18	13
144	18	10	18	18	13
148	16	10	18	18	7
152	15	9	17	18	6

Table S2 Workflow of candidate gene prioritization.

Ranking criterion	Database	Positive (+) or negative (-) influence on gene rank
(1) Cardiac expression and differential expression in the heart	Transcriptomes, this study	+
(2) Presence of a paralog in medaka (*Oryzias latipes*)	Ensembl	-
(3) Known cardiac phenotype in medaka (*Oryzias latipes*)	Ensembl	-
(4) Presence of a high confidence ortholog in zebrafish (*Danio rerio*)	Ensembl	+
(5) Known cardiac phenotype in zebrafish	Ensembl, ZFIN	-
(6) Known cardiac phenotype in mouse or rat	Ensembl, MGI/JAX	-
(7) Presence of high confidence ortholog in human	Ensembl	+
(8) Cardiovascular > ubiquitous expression	Ensembl, NCBI	+
(9) Evolutionary conservation:<br>(a) Presence of ortholog or (b) variant(s) associations in human	Ensembl	+

Table S3 Number (n) of injected and phenotyped embryos at 4 dpf. Surviving embryos were grouped into three categories: normal, cardiac affected and global phenotypes.

**Gene**	**sgRNA/crRNA**	**n injected**	**n normal**	**n cardiac**	**n global**	**n dead**	**Editor**
rrad	rrad_T1 sgRNA	93	18	35	20	20	CRISPR_Cas9
adprhl1	adprhl1_T1 sgRNA	81	25	13	17	26	CRISPR_Cas9
btbd1	btbd1_T1 sgRNA	100	50	19	21	10	CRISPR_Cas9
blzf1	blzf1_T1 sgRNA	84	38	15	14	17	CRISPR_Cas9
ptprd	ptprd_T1 sgRNA	54	22	10	8	14	CRISPR_Cas9
phka2	phka2_T1 sgRNA	62	14	12	7	29	CRISPR_Cas9
pcdh17	pcdh17_T1 sgRNA	92	49	16	12	15	CRISPR_Cas9
irf1	irf1a_T1 sgRNA	83	20	14	27	22	CRISPR_Cas9
gpc5a	gpc5a_T1 sgRNA	72	22	9	25	16	CRISPR_Cas9
gse1	gse1_T1 sgRNA	79	55	10	4	10	CRISPR_Cas9
sec61a1	sec61a1_T1 sgRNA	81	55	9	8	9	CRISPR_Cas9
zrsr2	zrsr2_T1 sgRNA	114	32	16	43	23	CRISPR_Cas9
rrad	rrad_T2 crRNA	75	21	19	15	20	evoBE4max
adprhl1	adprhl1_T2 crRNA	68	4	7	14	43	evoBE4max
btbd1	btbd1_T2 crRNA	53	16	10	10	17	evoBE4max
blzf1	blzf1_T2 crRNA	68	23	11	13	21	evoBE4max
ptprd	ptprd_T2 crRNA	54	27	8	0	19	evoBE4max
phka2	phka2_T2_crRNA	50	26	11	0	13	evoBE4max
mock_ctrl1	NA	41	36	1	0	4	NA_Cas9_ctrl
mock_ctrl2	NA	49	44	3	0	2	NA_Cas9_ctrl
mock_ctrl3	NA	46	42	0	2	2	NA_Cas9_ctrl
mock_ctrl4	NA	49	44	4	1	0	NA_Cas9_ctrl
mock_ctrl5	NA	48	42	3	2	1	NA_Cas9_ctrl
mock_ctrl6	NA	30	21	4	1	4	NA_Cas9_ctrl
mock_ctrl7	NA	40	28	2	2	8	NA_Cas9_ctrl
mock_ctrl1	NA	31	27	1	1	2	NA_BE_ctrl
mock_ctrl2	NA	46	34	0	3	9	NA_BE_ctrl
mock_ctrl3	NA	28	21	2	0	5	NA_BE_ctrl

Table S4 Knockout target sites. PAM sites specified in brackets.

Name	Sequence 5'-3'
adprhl1 T1 sgRNA	CTCTGGCGTAGATTTCCAAT[GGG]
adprhl1 T2 crRNA	ACAGCCCAAGCTCTCATAAC[AGG]
blzf1 T1 sgRNA	TCACGGCTGGGTGATTTGAC[TGG]
blzf1 T2 crRNA	GAGTCGAGAGGCCTGTACTG[AGG]
btbd1 T1 sgRNA	ACTTATGGGCCGGTATCCGC[TGG]
btbd1 T2 crRNA	CAGGCAGGCCCAGCGGATAC[CGG]
gpc5a T1 sgRNA	GATCTGGTTTATCACAGGAT[CGG]
gse1 T1 sgRNA	CGAAAGCATAGGGGTTTCCC[AGG]
irf1a T1 sgRNA	CGGGCTTGTCTTTACCCGGA[CGG]
pcdh17 T1 sgRNA	ATGCTTAGAGTTACAATAAG[AGG]
phka2 T1 sgRNA	GAACGACGTGGTTTGGACAC[TGG]
phka2 T2 crRNA	TGTCACCAGGTGAGTCCAGG[AGG]
ptprd T1 sgRNA	ATGAAGACCACAGCCAGCAC[AGG]
ptprd T2 crRNA	CTTTGAACCAAGAAATTTCC[GGG]
rrad T1 sgRNA	TCTGCGGTGTCGTTGCGCGC[AGG]
rrad T2 crRNA	GACACCGCAGACGGGACAAA[CGG]
sec61a1 T1 sgRNA	CTACTGGATGAGAGTAATAT[TGG]
zrsr2_T1 sgRNA	GAGCTTGTCGATTTGGAGAA[AGG]

Table S5 Oligonucleotides for CRISPR sgRNA cloning.

Name	Sequence 5'-3'
adprhl1_T1_ oligo_F	TAGGCTGGCGTAGATTTCCAAT
adprhl1_T1_ oligo_R	AAACATTGGAAATCTACGCCAG
blzf1_T1_ oligo_F	TAGGACGGCTGGGTGATTTGAC
blzf1_T1_ oligo_R	AAACGTCAAATCACCCAGCCGT
btbd1_T1_ oligo_F	TAGGTTATGGGCCGGTATCCGC
btbd1_T1_ oligo_R	AAACGCGGATACCGGCCCATAA
gpc5a_T1_ oligo_F	TAGGTCTGGTTTATCACAGGAT
gpc5a_T1_ oligo_R	AAACATCCTGTGATAAACCAGA
gse1_T1_ oligo_F	TAGGAAAGCATAGGGGTTTCCC
gse1_T1_ oligo_R	AAACGGGAAACCCCTATGCTTT
irf1a_T1_ oligo_F	TAGGGGCTTGTCTTTACCCGGA
irf1a_T1_ oligo_R	AAACTCCGGGTAAAGACAAGCC
pcdh17_T1_ oligo_F	TAGGGCTTAGAGTTACAATAAG
pcdh17_T1_ oligo_R	AAACCTTATTGTAACTCTAAGC
phka2_T1_ oligo_F	TAGGACGACGTGGTTTGGACAC
phka2_T1_ oligo_R	AAACGTGTCCAAACCACGTCGT
ptprd_T1_ oligo_R	AAACGTGCTGGCTGTGGTCTTC
ptprd_T1 oligo_F	TAGGGAAGACCACAGCCAGCAC
rrad_T1_ oligo_F	TAGGTGCGGTGTCGTTGCGCGC
rrad_T1_ oligo_R	AAACGCGCGCAACGACACCGCA
sec61a1_T1_ oligo_F	TAGGACTGGATGAGAGTAATAT
sec61a1_T1_ oligo_R	AAACATATTACTCTCATCCAGT
zrsr2_T1_ oligo_F	TAGGGCTTGTCGATTTGGAGAA
zrsr2_T1_ oligo_R	AAACTTCTCCAAATCGACAAGC

Table S6 Oligonucleotides used to PCR amplify the target loci.

Name	Sequence 5'-3'
ptprd_T1_F	CAGACCCACCCACGCTTC
ptprd_T1_R	TTCACACCCGCATACTTTGC
rrad_T1_F	GCATGGCTCATCTATCCACAGA
rrad_T1_R	TGTCTGTGTGGGATGTTTGGT
adprhl1_T1_F	ACCCAAACTTGTTGGAAGACAG
adprhl1_T1_R	CAGGAGAGCAGTCCACACAC
pcdh17_T1_F	GTTACTGGGGAGGTGCGAAC
pcdh17_T1_R	GCTCACCAAGGTCAGAGGTC
irf1a_T1_F	CATCAACCATTGAGTTGTCACA
irf1a_T1_R	TTCTGTGTTTGAGAGCGGGG
gpc5a_T1_F	TGCAGAGCTTTTATCAGAAACAGC
gpc5a_T1_R	TAAGATGGTTGCTGCAGGGG
btbd1_T1_F	AGGACCGATGTACAACTGGC
btbd1_T1_R	AGGTCAGTGACTTCCAGCTG
gse1_T1_F	AGAGGCTGCCACGTTACAAA
gse1_T1_R	GCTGTTGGTGCTCATTCCAG
sec61a1_T1_F	GTTTTCTTGAAACTGCAGATCCA
sec61a1_T1_R	AAAGCTTGTGAACTACAGATCAAA
zrsr2_T1_F	CACTTTGTTTATGAAGTCTGCGT
zrsr2_T1_R	GGGAAAAATTGCCTCGAAGTAA
blzf1_T1_F	AATGCAACATTCAGGACAGAACA
blzf1_T1_R	TGGAGCAGTTGAGTGTAACAGG
phka2_T1_F	GTCCAGTTGATGACGGCGAT
phka2_T1_R	GAGGACAGAGTGGTGTTTCA
adprhl1_T2_F	GTCCAATCAGCCTAGTTTGGT
adprhl1_T2_R	GTTGGGCTACAGGAAGGGAC
rrad_T2_F	GTCCCGTTTCCAGTCAACCT
rrad_T2_R	TCGTGACCGACATCCTCAAC
blzf1_T2_F	CTGTCTGCACTCGAAGCTGA
blzf1_T2_R	CCTATTCGGGGTCCTGGAGA
btbd1_T2_F	TAAGGGGTCCCGTCTACCTG
btbd1_T2_R	TCATGAAGCTGCGTCCAACT
phka2_T2_F	TCTGAGGATGCCAGGAGACC
phka2_T2_R	AAGATGTTTCAAACACAGTCTGGA
ptprd_T2_F	CAGCAGCAGCAAGTTCGAAA
ptprd_T2_R	CCTGACAGGTGGCATTGAGA

Movie S1

Swim tunnel assay of HdrR and HO5 individuals. Representative HdrR and HO5 adults in swim tunnel assay at 20m/s water flow. Note the steady swimming behavior at constant speed of the HdrR fish in contrast to the forward pushing and falling back of the HO5 individual during the 10 second movie (played in real time).

Movie S2

Embryonic heartbeat of candidate crispants. 10 second movies of embryonic heartbeat at 4 days post fertilization played in real time of unaffected control and *rrad*, *phka2*, *adprhl1*, *ptprd*, *blzf1, btbd1* crispants with cardiac phenotypes.

Movie S3

Embryonic heartbeat of candidate crispants with cardiac phenotype. 10 second movies of embryonic heartbeat at 4 days post fertilization played in real time of *irf1*, *zrsr2*, *gpc5a*, *pcdh17*, *gse1*, and *sec61a1* crispants with cardiac phenotypes.

Movie S4

Embryonic heartbeat of control and *rrad, blzf1 and adprhl1* F0 crispant (top row) and F2 mutants (bottom row). 10 second movies of embryonic heartbeat at 4 days post fertilization played in real time. Note the regular heartbeat in the control embryo versus the different degrees of atrioventricular (AV) block in the F0 crispants and F2 mutants.

Data S1

Associated fine mapped regions. Associated fine mapped regions across all temperatures 21°C, 28°C, and 35°C, as well as the variance phenotype (VA); selected candidate genes (green highlighted); see separate Excel file.

Data S2

Heart rate data functional validation. Raw data of heartbeat analysis (at 4 dpf) in crispants, editants and mock-injected embryos at 21 °C, 28 °C, and 35 °C; confer separate Excel file.

Data S3

Numbers of heart rate phenotyped embryos at 4 dpf for each temperature condition, see separate Excel file.

Data S4

Significance levels of heart rate differences at 21°C, 28°C., and 35°C. P values were assessed with the two-sided Wilcoxon test comparing the heart rate of the crispants and editants to the heart rate of the respective mock-injected control embryos., see separate Excel file.
