# Supplementary material for: Natural genetic variation quantitatively regulates heart rate and dimension
Source: Nat Commun. 2025 Apr 30;16:4062. doi: 10.1038/s41467-025-59425-7 (PMC12044080; doi:10.1038/s41467-025-59425-7)
Supplement: Supplementary file 2 — Description of Additional Supplementary Files [file 41467_2025_59425_MOESM2_ESM.pdf]

Description of Additional Supplementary Files

Movie S1
Swim tunnel assay of HdrR and HO5 individuals. Representative HdrR and HO5 adults in swim tunnel assay at 20m/s water flow. Note the steady swimming behavior at constant speed of the HdrR fish in contrast to the forward pushing and falling back of the HO5 individual during the 10 second movie (played in real time).

Movie S2
Embryonic heartbeat of candidate crispants. 10 second movies of embryonic heartbeat at 4 days post fertilization played in real time of unaffected control and rrad, phka2, adprhl1, ptprd, blzf1, btbd1 crispants with cardiac phenotypes.

Movie S3
Embryonic heartbeat of candidate crispants with cardiac phenotype. 10 second movies of embryonic heartbeat at 4 days post fertilization played in real time of irf1, zrsr2, gpc5a, pcdh17, gse1, and sec61a1 crispants with cardiac phenotypes.

Movie S4
Embryonic heartbeat of control and rrad, blzf1 and adprhl1 F0 crispant (top row) and F2 mutants (bottom row). 10 second movies of embryonic heartbeat at 4 days post fertilization played in real time. Note the regular heartbeat in the control embryo versus the different degrees of atrioventricular (AV) block in the F0 crispants and F2 mutants.

Data S1
Associated fine mapped regions. Associated fine mapped regions across all temperatures 21°C, 28°C, and 35°C, as well as the variance phenotype (VA); selected candidate genes (green highlighted); see separate Excel file.

Data S2
Heart rate data functional validation. Raw data of heartbeat analysis (at 4 dpf) in crispants, editants and mock-injected embryos at 21 °C, 28 °C, and 35 °C; confer separate Excel file.

Data S3
Numbers of heart rate phenotyped embryos at 4 dpf for each temperature condition, see separate Excel file.

Data S4
Significance levels of heart rate differences at 21°C, 28°C., and 35°C. P values were assessed with the two-sided Wilcoxon test comparing the heart rate of the crispants and editants to the heart rate of the respective mock-injected control embryos., see separate Excel file.
