# Supplementary material for: Natural genetic variation quantitatively regulates heart rate and dimension
Source: Nat Commun. 2025 Apr 30;16:4062. doi: 10.1038/s41467-025-59425-7 (PMC12044080; doi:10.1038/s41467-025-59425-7)
Supplement: Supplementary file 11 — Reporting Summary [file 41467_2025_59425_MOESM11_ESM.pdf]

nature portfolio

Corresponding author(s): Joachim Wittbrodt

Last updated by author(s): Mar 25, 2025

Reporting Summary

Nature Portfolio wishes to improve the reproducibility of the work that we publish. This form provides structure for consistency and transparency in reporting. For further information on Nature Portfolio policies, see our Editorial Policies and the Editorial Policy Checklist.

Statistics

For all statistical analyses, confirm that the following items are present in the figure legend, table legend, main text, or Methods section.

n/a	Confirmed	
☐	☒	The exact sample size (*n*) for each experimental group/condition, given as a discrete number and unit of measurement
☐	☒	A statement on whether measurements were taken from distinct samples or whether the same sample was measured repeatedly
☐	☒	The statistical test(s) used AND whether they are one- or two-sided<br>*Only common tests should be described solely by name; describe more complex techniques in the Methods section.*
☐	☒	A description of all covariates tested
☐	☒	A description of any assumptions or corrections, such as tests of normality and adjustment for multiple comparisons
☐	☒	A full description of the statistical parameters including central tendency (e.g. means) or other basic estimates (e.g. regression coefficient) AND variation (e.g. standard deviation) or associated estimates of uncertainty (e.g. confidence intervals)
☐	☒	For null hypothesis testing, the test statistic (e.g. *F*, *t*, *r*) with confidence intervals, effect sizes, degrees of freedom and *P* value noted<br>*Give P values as exact values whenever suitable.*
☒	☐	For Bayesian analysis, information on the choice of priors and Markov chain Monte Carlo settings
☒	☐	For hierarchical and complex designs, identification of the appropriate level for tests and full reporting of outcomes
☒	☐	Estimates of effect sizes (e.g. Cohen's *d*, Pearson's *r*), indicating how they were calculated

Our web collection on statistics for biologists contains articles on many of the points above.

Software and code

Policy information about availability of computer code

Data collection	Acquifer Imaging Machine for automated microscopy; Stereo microscope (Nikon SMZ18 equipped with a DS-Fi2 camera), Sp8 Confocal microscope (Leica)
Data analysis	HeartBeat software (Gierten, J. et al. Automated high-throughput heartbeat quantification in medaka and zebrafish embryos under physiological conditions. Sci Rep 10), R, Fiji,<br><br>https://github.com/tf2/ViteRbi

For manuscripts utilizing custom algorithms or software that are central to the research but not yet described in published literature, software must be made available to editors and reviewers. We strongly encourage code deposition in a community repository (e.g. GitHub). See the Nature Portfolio guidelines for submitting code & software for further information.

Data

Policy information about availability of data

All manuscripts must include a data availability statement. This statement should provide the following information, where applicable:

- Accession codes, unique identifiers, or web links for publicly available datasets
- A description of any restrictions on data availability
- For clinical datasets or third party data, please ensure that the statement adheres to our policy

All data supporting the findings of this study are available within the paper and its Supplementary Information. Source data are provided with this paper (data S1-S4).
Raw data are available upon request to the corresponding authors.
The individual raw sequencing datasets are linked to the following project IDs:
Illumina DNA sequencing data (PRJEB17699)
https://www.ebi.ac.uk/ena/browser/view/PRJEB17699
Illumina RNA sequencing data (PRJEB43091)
https://www.ebi.ac.uk/ena/browser/view/PRJEB43091

Research involving human participants, their data, or biological material

Policy information about studies with human participants or human data. See also policy information about sex, gender (identity/presentation), and sexual orientation and race, ethnicity and racism.

Reporting on sex and gender	n.a.
Reporting on race, ethnicity, or other socially relevant groupings	n.a.
Population characteristics	n.a.
Recruitment	n.a.
Ethics oversight	n.a.

Note that full information on the approval of the study protocol must also be provided in the manuscript.

Field-specific reporting

Please select the one below that is the best fit for your research. If you are not sure, read the appropriate sections before making your selection.

☒ Life sciences ☐ Behavioural & social sciences ☐ Ecological, evolutionary & environmental sciences

For a reference copy of the document with all sections, see nature.com/documents/nr-reporting-summary-flat.pdf

Life sciences study design

All studies must disclose on these points even when the disclosure is negative.

Sample size	Power analysis was undertaken using simulation where we considered the size of the medaka genome, likely number of recombinations per chromosome and variant effect size distribution to determine the optimum number of F2 samples needed for locus mapping.
Data exclusions	Datasets with very low sequencing coverage were excluded from data analysis and datasets with insufficient heart resolution were not considered.
Replication	different gene editing tools were used in distinct experimental series to assess functional consequences.
Randomization	not relevant to our study.
Blinding	F2 phenotyping was blinded during data collection and heart rate analysis - genotypes were correlated afterwards.

Reporting for specific materials, systems and methods

We require information from authors about some types of materials, experimental systems and methods used in many studies. Here, indicate whether each material, system or method listed is relevant to your study. If you are not sure if a list item applies to your research, read the appropriate section before selecting a response.

Materials & experimental systems

n/a	Involved in the study	
☐	☒	Antibodies
☒	☐	Eukaryotic cell lines
☒	☐	Palaeontology and archaeology
☐	☒	Animals and other organisms
☒	☐	Clinical data
☒	☐	Dual use research of concern
☒	☐	Plants

Methods

n/a	Involved in the study	
☒	☐	ChIP-seq
☒	☐	Flow cytometry
☒	☐	MRI-based neuroimaging

Antibodies

Antibodies used

myl7 primary antibody (Genetex, Catalog no. GTX128346-200)

Validation

Validation experiments are mentioned on manufacturer's website wherein myl stains for myosin light chain in zebrafish and medaka. For details compare: https://www.genetex.com/Product/Detail/Myl7-antibody/GTX128346?srsltid=AfmBOooya4FDLKQMdhcjh_gKPDjOapJEz|6cDjT43Gz3CT_OJMuL5FjM.

Animals and other research organisms

Policy information about studies involving animals; ARRIVE guidelines recommended for reporting animal research, and Sex and Gender in Research

Laboratory animals

The wild-type Kaga, HNI, Cab, HO5 and HdrR strain and a fluorescent cardiac reporter line HdrR (myl7::EGFP myl7::H2A-mCherry) were used in this study. All animals were kept as closed stocks in the approved facility of the Wittbrodt lab at COS Heidelberg.

Wild animals

n.a.

Reporting on sex

Heart and liver samples were dissected from adult HO5 and HdrR female fish.

Field-collected samples

n.a.

Ethics oversight

Regierungspräsidium Karlsruhe.

Note that full information on the approval of the study protocol must also be provided in the manuscript.

Plants

Seed stocks

n.a.

Novel plant genotypes

n.a.

Authentication

n.a.
